# Effect of CTSS non-synonymous mutations on litter size in Qianbei Ma goats

**DOI:** 10.3389/fvets.2023.1276673

**Published:** 2023-11-27

**Authors:** Yuan Zhang, Xiang Chen, Yong Ruan, Wei Guo, Jiajing Chen, Wen Tang, Quan Ji, Kaibin Fu

**Affiliations:** ^1^Key Laboratory of Animal Genetics, Breeding and Reproduction in the Plateau Mountainous Region, Ministry of Education, Guizhou University, Guiyang, China; ^2^Key Laboratory of Animal Genetics, Breeding and Reproduction of Guizhou Province, Guizhou University, Guiyang, China; ^3^College of Animal Science, Guizhou University, Guiyang, China

**Keywords:** non-synonymous mutation, litter size, *CTSS* gene, single nucleotide polymorphism, goat

## Abstract

Cathepsin S (CTSS) is a member of the cysteine protease family closely related to reproductive regulation in goats. However, its effect on litter size in goats remains unclear. In this study, the relationship between *CTSS* gene polymorphisms and litter size was revealed by analyzing the DNA sequence and mRNA expression of *CTSS* in the gonadal axis of Qianbei Ma goats. In addition, bioinformatics methods were used to evaluate the effect of non-synonymous mutations on CTSS protein structure and function. *CTSS* was expressed in all parts of the gonadal axis of Qianbei Ma goats, with the highest expression in the uterus in the multi-lamb group and in the fallopian tube in the single-lamb group. The sequencing results showed that four SNPs in *CTSS*, including g.7413C → T, g.8816A → T, g.9191 T → G and g.10193G → A, were significantly correlated with litter size (*p* < 0.05). All four analyzed mutation sites were in strong linkage disequilibrium (*r*^2^ > 0.33, D′ > 0.70). Additionally, the haplotype Hap1/2 had a significantly higher frequency than the other haplotypes (*p* < 0.05). g.7413C → T and g.8816A → T were non-synonymous mutations. The g.7413C → T mutation resulted in the substitution of serine 161 of the CTSS protein with phenylalanine (p.S161F), and the g.8816A → T mutation resulted in the substitution of aspartate 219 with tyrosine (p.N219Y). p.S161F was highly conserved across 13 species and that p.N219Y was relatively conserved in cloven-hoofed species. Mutations at two sites changed the local conformation of the CTSS protein, reduced its stability, and affected its function and goat breed evolution. These findings confirm that *CTSS* affects the lambing traits of goats and provide a theoretical basis for the regulatory mechanism of *CTSS* in affecting litter size.

## 1 Introduction

The cathepsin (CTS) family of enzymes is widely expressed in various cells and tissues (1), playing important roles in catalyzing protein hydrolysis and regulating various normal biological processes such as cell death, proliferation, migration, cancer development and processing of antigens and antibodies (2, 3). The CTS family contains a variety of subtypes. Cathepsins mainly include cysteine cathepsins, serine cathepsins (cathepsins A, G) and aspartic cathepsins (cathepsins D, Sand E) (4). Activation of pregnancy-specific lysosomal function by CTS in blood leukocytes is highly correlated with interferon-τ (IFNT) expression during maternal–fetal recognition of pregnancy in pregnant cows (5). Cathepsin S (CTSS) is a lysosomal cysteine protease (6). Previous studies on this gene have focused on its role in the immune response, inflammatory response, cardiovascular disease progression and tumor progression (7–9). The activity of this gene is also closely associated with fibronectin degradation and obesity (10). *CTSS* regulates the secretion of progesterone and estradiol and the proliferation and apoptosis of ovarian granulosa cells in rabbits and is closely related to the regulation of early gestation in goats (11, 12). However, the mechanism underlying the effect of *CTSS* on litter size in goats is unclear.

Qianbei Ma goats are a unique goat breed raised in the Guizhou Plateau Mountain area of China. This breed is characterized by early sexual maturity, good adaptability, strong disease resistance and stable genetic performance (13) However, its low reproduction rate is a constraint to the development and utilization of this species. Litter size is an important index for quantifying the reproductive performance of female livestock (14). The average lambing rate of Qianbei Ma goats is approximately 207%. However, the breed comprises three groups: high reproductive rate, low reproductive rate and sterile (15). The low heritability of lambing traits in goats limits the traditional methods of selection for high reproductive performance groups. Therefore, it is important to study the expression of *CTSS*-encoding genes in the Qianbei Ma goat population in order to understand the relationship between *CTSS* gene polymorphisms and lambing traits and to screen for molecular markers associated with lambing traits to guide the breeding of Qianbei Ma goats.

## 2 Materials and methods

### 2.1 Experimental animals

The animals used in this study strictly comply with the guidelines of the Animal Welfare Committee of Guizhou University (EAE-GZU-2022-E030, 25th October, 2022). The Qianbei Ma goats used in the study were obtained from Fuxing Herding Co Ltd., Xishui County, Guizhou Province, China. Hundred and sixty healthy Qianbei Ma ewes with similar body weights were selected and the total number of births and live births of the first, second and third fetuses of the group were recorded. Four milliliters of blood were drawn through the jugular vein into EDTA anticoagulation tubes and stored in a refrigerator at −20°C. Three singleton pregnancy and three multiple pregnancy ewes were selected from 160 Qianbei Ma goats for which reproductive data were recorded, and were euthanized by carotid artery bloodletting after electrocution. Goat gonadal axis tissue samples (including hypothalamus, pituitary, ovaries, uterus and fallopian tubes) were collected within 20 min of euthanasia and washed with phosphate buffered saline solution (PBS). All samples were then rapidly frozen in liquid nitrogen and subsequently transferred to a − 80°C freezer for storage.

### 2.2 RNA and DNA extraction and cDNA synthesis

Total RNA was extracted from the gonadal axis tissues of the singleton pregnancy and multiple pregnancy groups using TRIzol reagent (Invitrogen, Carlsbad, CA, USA) and a RNeasy RNA purification kit containing DNase treatment (Qiagen, Valencia, CA, USA) according to the manufacturer’s instructions. DNA extraction from the collected blood was performed strictly according to the instructions of the Blood DNA Extraction Kit (Beijing Tiangen Biochemical Technology Co., Ltd., Beijing, China). The concentration and purity of the RNA and DNA were measured using an ultramicro ultraviolet spectrophotometer (NanoDrop2000; Thermo Scientific, Waltham, MA, USA). The samples were tested for integrity on 1% agarose gels, and all samples were stored in a −20°C refrigerator.

### 2.3 Primer design and synthesis

According to the RNA (accession number: XM_005677657) and DNA (accession number: NC_030810.1) sequences of goat *CTSS* as published in NCBI, Primer Premier 5.0 (PREMIER Biosoft International, Palo Alto, CA, USA) was used to design primers for amplification. Using β-actin as a fluorescent quantitative internal reference gene, primer sequences were sent to Beijing Tsingke Biotechnology Co., Ltd. for synthesis (Chongqing, China). The primer sequence information is shown in Table 1.

**Table 1:** Primer information.

Genes	Primer sequence (5′ → 3′)	Product size/bp	Annealing temperature∕°C
CTSS-Exon1,2	F:5’ AGGAAATCACGGAGGAAACCAG 3′ R:5’ CCTCAGGATTGAAATATTCAAGCC 3’	639	63
CTSS-Exon3	F:5’ GTAAAGTCCCTGCTTCCCTCAT 3′ R:5’ CCAGGCTCCTATACTATCCATGAA 3’	555	63
CTSS-Exon4	F:5’ AGAGGAAGAGTTAAGATTGGTGTGC 3′ R:5’ GGAAAGTGGTCACAGTGTAGATCAA 3’	483	63
CTSS-Exon5	F:5’ TCTTCTCCTTCCCGATGTCTGA 3′ R:5’ CCTAAGGGACTATGAGATTCACTGC 3’	457	59
CTSS-Exon6	F:5’ ATTAAAGTTAGACCTTGTTCCGGAG 3′ R:5’ CGGCTTGGTGATAAGTTTAGTACAG 3’	495	61
CTSS-Exon7	F:5’ TCCTCCGTTACTGGTGAAACATAG 3′ R:5’ ACACAACTGAACAACAAGCACACA 3’	642	63
CTSS-Exon8	F:5’ ATAGCATTGAGGGCAAAGAACC 3′ R:5’ CTTATTGCTTGATTAGTTCTGGAGG 3’	478	63
CTSS-Exon9	F:5’ CTCATTCTATGCAGAAGCAGGAGG 3′ R:5’ TAATCTGGAGCAGGTGTGAGGAATA 3’	1,160	63
q-CTSS	F:5’ AAGTAGCACGGCGTCTCAT 3′ R:5’ TGTCTCCCAGGTGGTTCAT 3’	114	58
β-actin	F:5’ TGATATTGCTGCGCTCGTGGT 3′ R:5’ GTCAGGATGCCTCTCTTGCTC 3’	189	58

### 2.4 PCR amplification and real-time fluorescence quantitative PCR analysis

Total PCR amplification system (20 μL): 10 μL 2× Taq PCR Master Mix (Beijing Tsingke Biological Co., Ltd., Beijing, China), 1 μL DNA template, 1 μL each forward and reverse primers (10 μmol/L), 7 μL deionized water (ddH2O). The PCR procedure was as follows: predenaturation at 98°C for 3 min, denaturation at 98°C for 10 s, annealing at 60°C for 10 s, and extension at 72°C for 15 s. After 35 cycles, the samples were stored at 4°C. After the PCR amplification products were tested by 1% agarose gel electrophoresis to check the expected fragment size, the PCR amplification products were sent to a biological company for sequencing.

The reaction system for fluorescence quantitative PCR (10 μL) contained 5 μL of 2 × UltraSYBR Mixture (Beijing Tsingke Biotechnology Co., Ltd., Beijing, China), 0.5 μL of cDNA, 0.5 μL each of the forward and reverse primers, and ddH2O to 10 μL. The reaction conditions were as follows (see Table 1 for details): 1 cycle at 95°C for 2 min, followed by 40 cycles at 95°C for 15 s, at the appropriate annealing temperature for 30 s, and at 72°C for 30 s. The melting curve was generated automatically by the machine (base temperature 65°C, increasing by 0.5°C every 5 s to 95°C). The annealing temperature of β-actin was the same as the annealing temperature for each experimental gene. The specificity of the PCR primers was confirmed by the presence of a single peak in the melting curve. Three biological replicates were established for each sample.

### 2.5 Bioinformatics analysis

Evaluation of sequencing results and analysis of polymorphic loci in *CTSS* were performed using SeqMan and MegAlign in DNAStar (16, 17). Effect of non-synonymous single-nucleotide polymorphisms (nsSNPs) on CTSS protein function were predicted using PhD-SNP and SNAP2 (18, 19). I-Mutant 2.0 and MuPro were used to predict the effect of nsSNPs on protein stability (20, 21). Generation of multiple sequence comparisons based on CTSS amino acid sequences was performed using Cluster Omega for assessing CTSS sequence conservation (22). Sopma was used to analyze the secondary structure of CTSS proteins and AlphaFold2 was used to assess the tertiary structure of wild-type and mutant CTSS proteins (23–25).

### 2.6 Statistical analysis

The sequencing results were analyzed, the peaks were plotted against one other using SeqMan software, and the identified SNP loci were analyzed statistically. The 2^-ΔΔCt^ method was used to calculate the differential expression levels of the *CTSS* gene in the ovary, uterus, fallopian tube, pituitary and hypothalamus, and then the expression level of *CTSS* mRNA in tissues was analyzed by GraphPad Prism 6. Allele frequencies and genotype frequencies were calculated using Haploview 4.2. Population genetic indicators such as polymorphism information content (PIC), gene purity (Ho), effective allele number (Ne), and gene heterozygosity (He) were analyzed according to Chakraborty and Nei (26). Linkage disequilibrium (LD) analysis and haplotype analysis of SNP loci in *CTSS* were performed using the SHEsis platform.

The experimental data of different genotypes were analyzed using one-way ANOVA in PASW Statistics 18 software to identify associations between different genotypes and reproductive performance, and the analyzed data are expressed as the means ± standard deviations.

## 3 Results

### 3.1 Expression profile of *CTSS* in the gonadal axis

**Figure 1:**
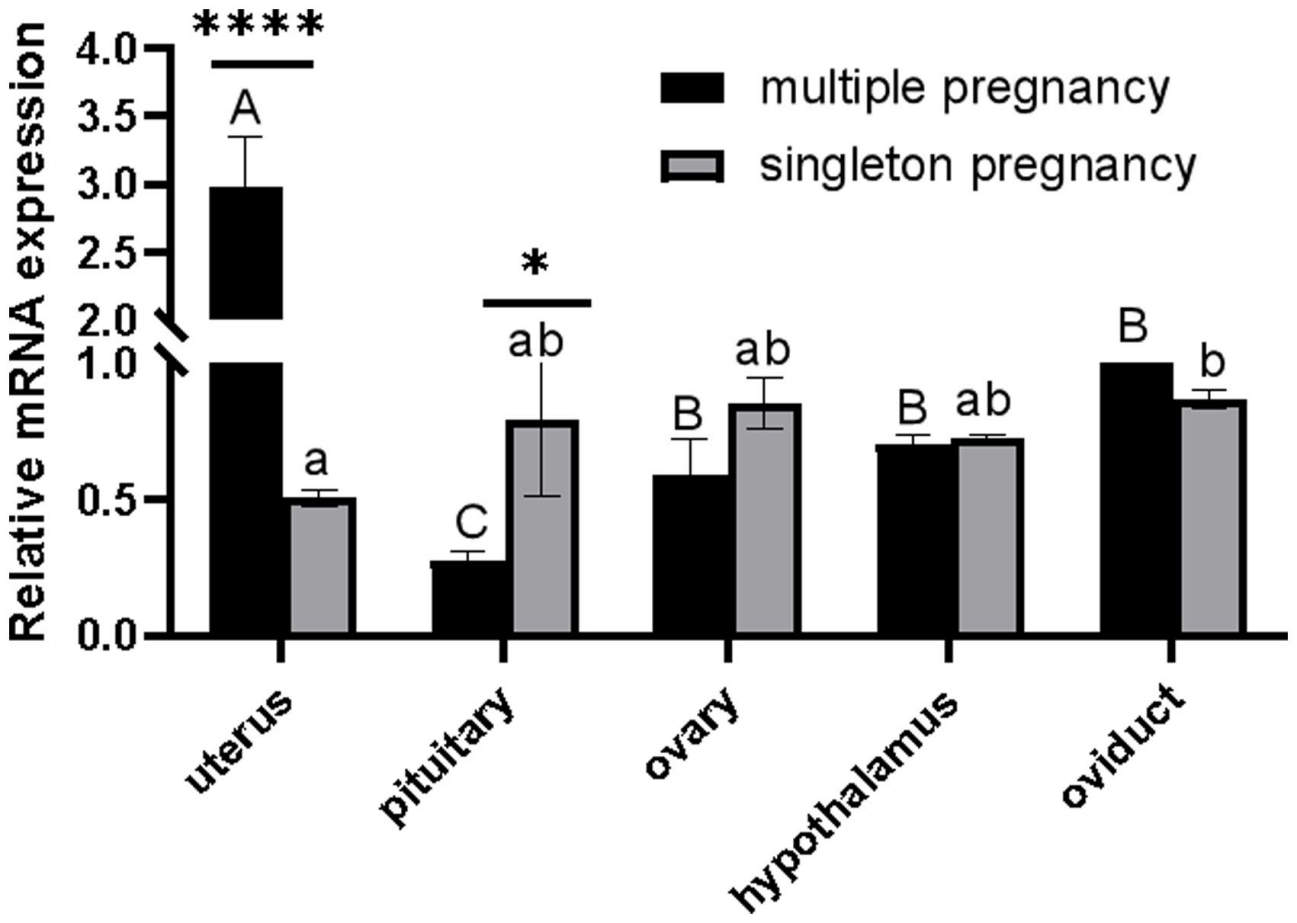
Differential expression analysis of CTSS gene in the gonadal axis of single and multi-lamb Qianbei Ma goat. "****" indicated that there were very significant differences among the same tissues of single and multi-lamb ewe (*P* < 0.0001). "A, B, C" means very significant difference between different tissues of multi-lamb ewes (*P* < 0.01), "a, b" means significant difference between different tissues of singletons ewes (*P* < 0.05), and the same letter means no significant differ.

As shown in Figure 1, *CTSS* mRNA expression in the gonadal axis of the multi-lamb ewe population was significantly higher in the uterus than in other gonadal tissues (*p* < 0.01) and was significantly lower in the pituitary gland than in the ovary, hypothalamus and oviduct (*p* < 0.05). Analysis between the singleton pregnancy and multiple pregnancy groups showed that *CTSS* gene expression was significantly higher in the uterus of multiple pregnancy ewes than in the uterus of singleton pregnancy ewes (*p* < 0.01), and it was significantly higher in the pituitary gland of the single-lambing ewes group than in the pituitary gland of the multilambing ewes (*p* < 0.05); the expression of *CTSS* was similar among the remaining tissues, indicating that *CTSS* plays an important role in the regulation of lambing traits.

### 3.2 PCR amplification

**Figure 2:**
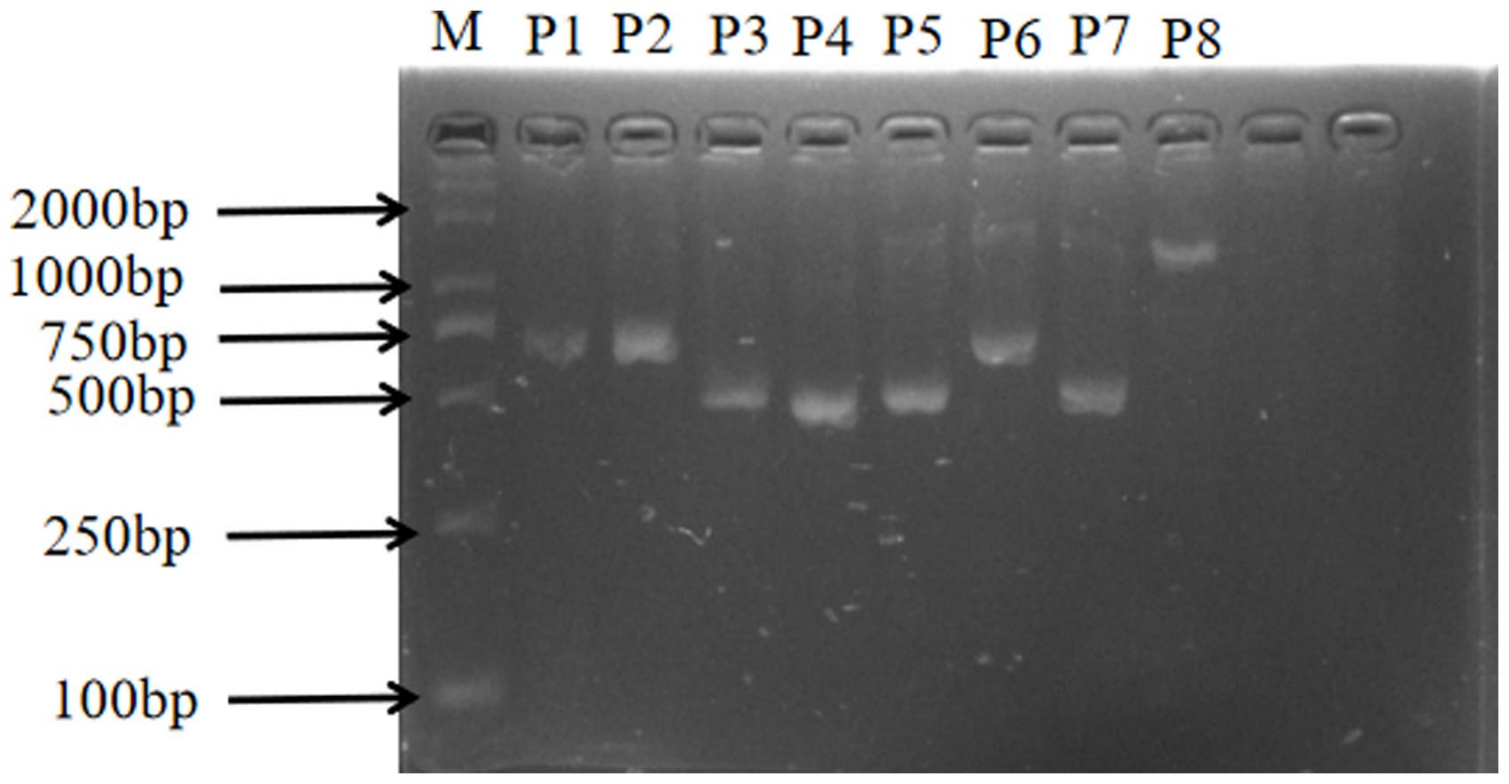
*CTSS* gene amplification results. M: DL-2000Marker; P1: exon1 and exon2; P2-P8: exon 2 – exon 8.

In this study, the sizes of the PCR amplification products were consistent with the expected fragment sizes, and the bands appeared clear and bright, with no specific amplification and no obvious trailing phenomenon, confirming the good primer specificity and the ability to be used for direct sequencing (Figure 2).

### 3.3 *CTSS* gene polymorphism analysis

**Figure 3:**
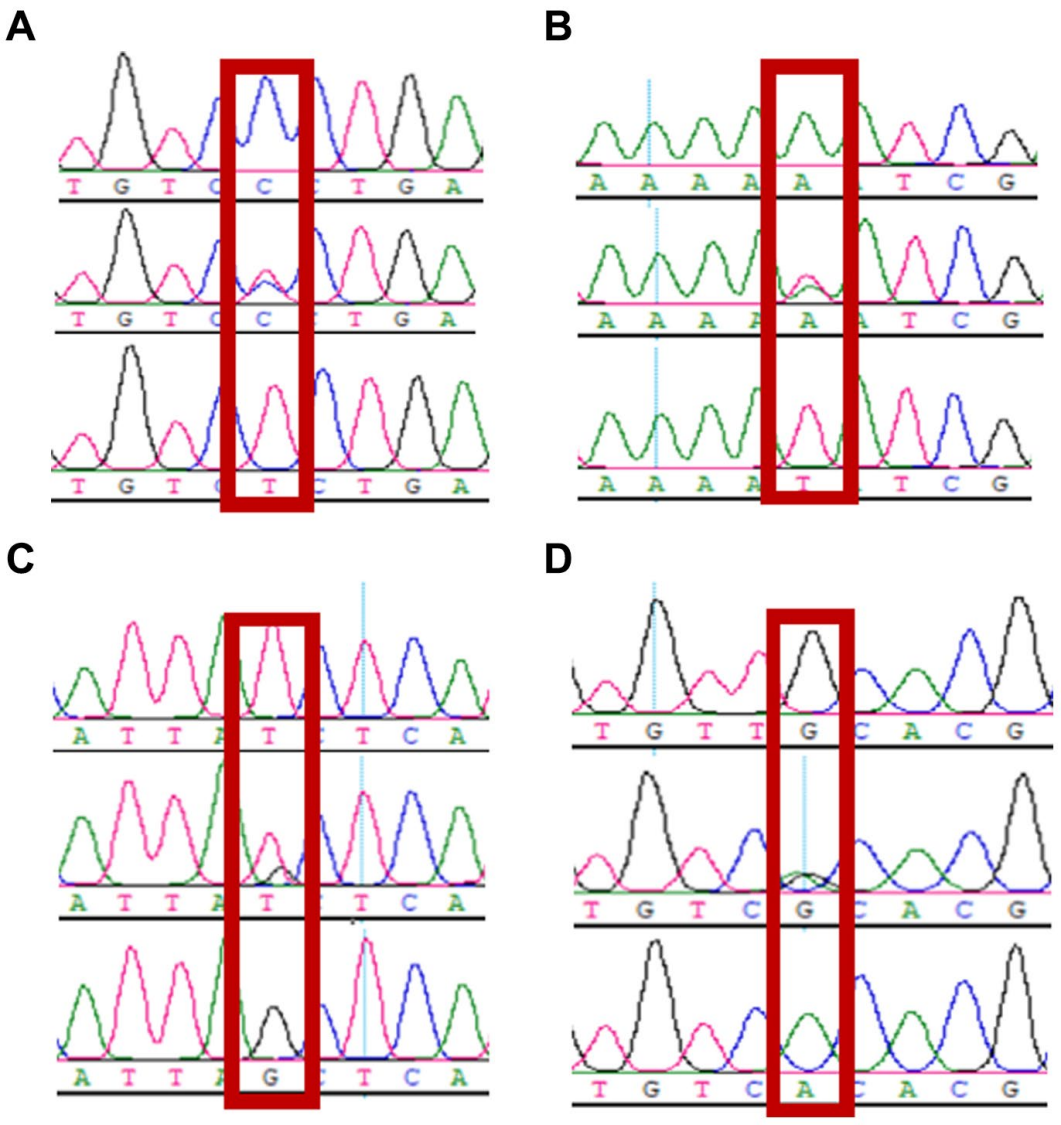
Sequencing peaks of 4 snp of *CTSS* gene (*n* = 160). **(A)** g.7413C → T, **(B)** g.8816 A → T, **(C)** g.9191 T → G, **(D)** g.10193 G → A.

The sequencing data were aligned against the *CTSS* (NC_030810.1) reference sequence using DNAStar software. Two SNP loci, g.7413C → T (exon 6) and g.8816A → T (exon 7), were identified in the exon 6 and 7 regions of the *CTSS* gene; two SNP loci, g.9191 T → G (intron 7) and g.10193G → A (intron 8), were found in the intron 7 and 8 regions. All of the four SNP loci listed above were present with two alleles and resulted in three genotypes. The sequencing chromatograms are shown in Figure 3.

**Figure 4:**
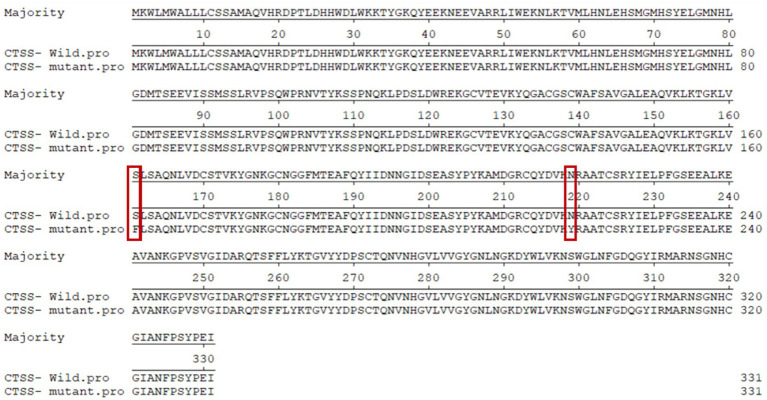
Amino acid sequence comparison of wild-type and mutant CTSS. Red boxes indicate non-synonymous SNPs.

The identified sequences were aligned to GenBank reference sequences using MegAlign and compared using the Clustal W method. g.7413C → T is the non-synonymous nsSNP leading to the substitution of serine with phenylalanine, and g.8816A → T is the nsSNP leading to the substitution of aspartic acid with tyrosine; g.9191 T → G and g.10193G → A are synonymous mutations (Figure 4).

### 3.4 Population genetic analysis of *CTSS*

The four SNP loci were genetically characterized, and all four mutant loci had three genotypes. By chi-square test (χ^2^), the genotype distributions of g.7413C → T, g.8816A → T and g.10193G → A did not deviate from Hardy–Weinberg equilibrium (*p* > 0.05), while g.9191 T → G deviated from HWE (*p* < 0.05) (Table 2).

**Table 2:** Genotype frequencies and gene frequencies of *CTSS* in Qianbei Ma goat.

SNPs	Genotype frequency			Gene frequency		χ^2^	*P*
g.7413\u00B0C → T	CC	CT	TT	C	T	0.847	0.357
	0.081(13)	0.356(57)	0.563(90)	0.259	0.741		
g.8816 A → T	AA	AT	TT	A	T	3.039	0.081
	0.094(15)	0.331(53)	0.575(92)	0.259	0.741		
g.9191 T → G	TT	TG	GG	T	G	4.444	0.035
	0.094(15)	0.312(50)	0.594(95)	0.25	0.75		
g.10193 G → A	GG	GA	AA	G	A	3.441	0.064
	0.081(13)	0.306(49)	0.613(98)	0.234	0.766		

χ^2^_0.05_ < 5.99; χ^2^_0.01_ < 9.21, *P* > 0.05 indicates that the population is in Hardy–Weinberg equilibrium, with sample size in parentheses.

The effective number of alleles per SNP in the *CTSS* ranged from 1.560 to 1.624, the heterozygosity ranged from 0.359 to 0.384, and the purity ranged from 0.616 to 0.641 (Table 3). The polymorphism level in Qianbei Ma goats was intermediate, ranging from 0.295 to 0.310, indicating that the polymorphic loci were rich in genetic information.

**Table 3:** Genetic diversity of *CTSS* gene SNPs loci in Qianbei Ma goat.

SNPs	Effective allele numbers (Ne)	Heterozygosity (He)	Homozygosity (Ho)	Polymorphism information content (PIC)
g.7413C → T	1.624	0.384	0.616	0.310
g.8816 A → T	1.624	0.384	0.616	0.310
g.9191 T → G	1.600	0.375	0.625	0.305
g.10193 G → A	1.560	0.359	0.641	0.295

PIC < 0.25 means low polymorphism, 0.25 < PIC < 0.50 means moderate polymorphism, PIC > 0.5 means high polymorphism.

### 3.5 LD and haplotype analyses of *CTSS* gene SNPs

**Figure 5:**
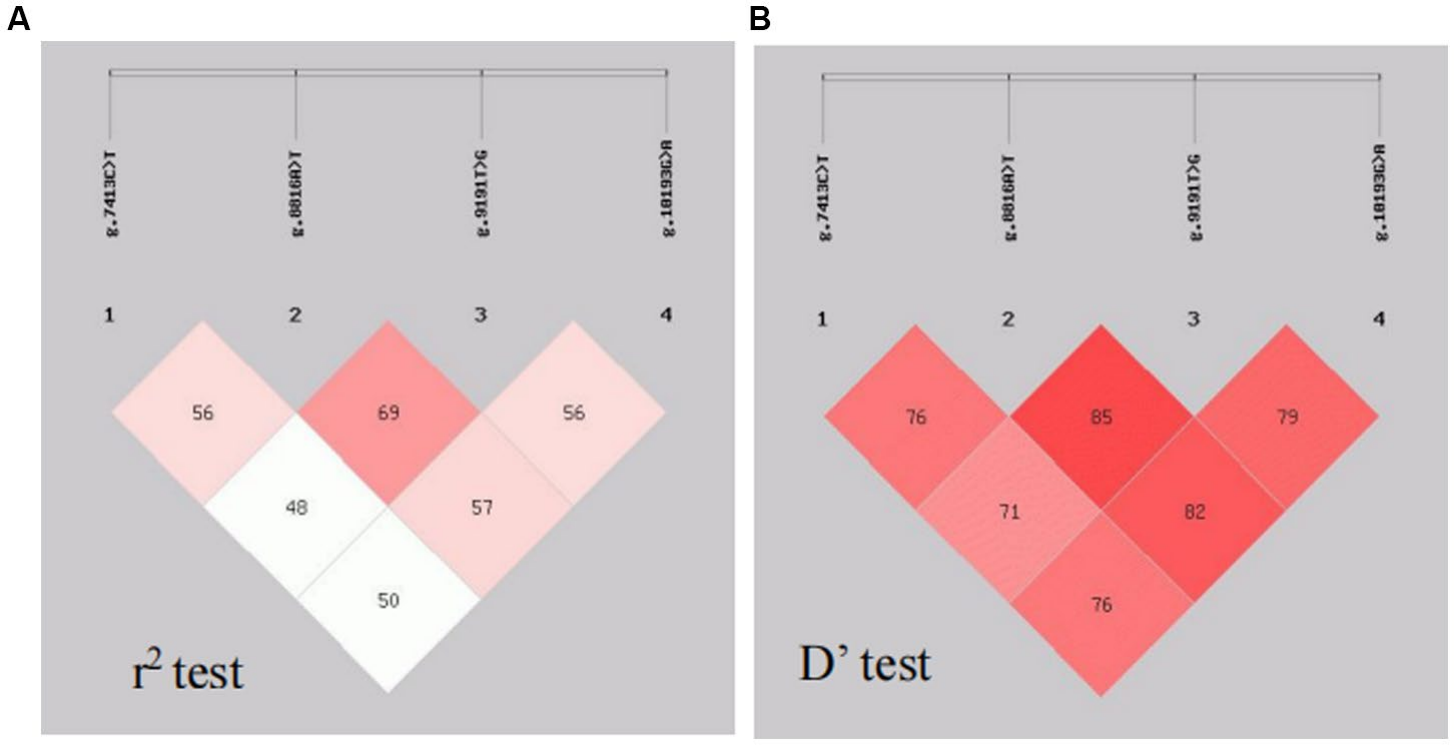
Linkage disequilibrium analysis. The SPNsg.7413C → T, g.8816A → T, g.9191T → G and g.10193G → A show strong LD (r 2 > 0.33, D’ > 0.70).

Analysis of LD was performed with the four SNPs of the CTSS gene. The results are shown in Figure 5. The SNP loci g.7413C → T, g.8816A → T, g.9191 T → G and g.10193G → A show strong LD (r^2^ > 0.33, D′ > 0.70).

Using the SHEsis platform, 2 CTSS haplotypes were identified: Hap1 (−TTGA-) and Hap2 (-CATG-); haplotypes with frequencies <3.% were not involved in the analysis. Hap2 had the highest frequency, accounting for 68.5% of all haplotypes, followed by Hap1 with 17.0% (Table 4). Random combination of two haplotypes produced three diploids, Hap1/1, Hap1/2 and Hap2/2.

**Table 4:** *CTSS* gene haplotypes and frequencies.

Haplotype	g.7413C>T	g.8816 A>T	g.9191 T>G	g.10193 G>A	Frequency
Hap1	T	T	G	A	0.170
Hap2	C	A	T	G	0.685

### 3.6 Relationship between *CTSS* polymorphisms and litter size

Association analysis of the *CTSS* gene SNP loci combined with the number of lambs produced in litters 1–3 of Qianbei Ma ewes was performed, and the results are shown in Table 5. The g.7413C → T, g.8816A → T, g.9191 T → G and g.10193G → A SNP loci were significantly correlated with the number of lambs born to Qianbei Ma goats. The frequency of the g.7413 TT genotype was significantly higher than that of CC and CT genotypes at the C → T locus in second births; the frequency of the g.8816 TT genotype was significantly higher than that of the AA genotype at the A → T locus in second births; the frequency of the g.9191 GG genotype was significantly higher than that of the TT genotype at the T → G locus in second births and the frequency of the TG genotype was significantly higher than that of the GG genotype in third births; the frequency of the g.10193 AA genotype was significantly higher than that of the GG genotype at the G → A locus in first births (all *p* < 0.05).

**Table 5:** Association analysis between *CTSS* gene polymorphism and the number of lambs produced in 1 ~ 3 litters of Qianbei sheep.

SNPs	Genotype	First-born	Second-born	Third-born
g.7413C → T	CC	1.846 ± 0.375	1.846 ± 0.533b	2.078 ± 0.494
	CT	2.035 ± 0.597	2.070 ± 0.529b	2.230 ± 0.732
	TT	2.089 ± 0.466	2.289 ± 0.503a	2.200 ± 0.690
g.8816 A → T	AA	1.867 ± 0.352	1.933 ± 0.458b	2.133 ± 0.516
	AT	1.981 ± 0.604	2.094 ± 0.628ab	2.226 ± 0.669
	TT	2.054 ± 0.427	2.315 ± 0.490a	2.174 ± 0.689
g.9191 T → G	TT	1.867 ± 0.352	2.000 ± 0.378b	2.133 ± 0.516ab
	TG	1.980 ± 0.622	2.040 ± 0.605ab	2.320 ± 0.683a
	GG	2.063 ± 0.480	2.290 ± 0.523a	2.116 ± 0.666b
g.10193 G → A	GG	1.769 ± 0.439b	2.000 ± 0.577	2.231 ± 0.599
	GA	2.000 ± 0.646ab	2.163 ± 0.553	2.225 ± 0.715
	AA	2.061 ± 0.450a	2.214 ± 0.561	2.153 ± 0.648

Data in the table are compared in the same column within the same locus, different letters indicate significant differences between genotypes (P < 0.05) and the same letters indicate non-significant differences (*P* > 0.05).

Association analysis of diploidy with the number of lambs born in litters 1–3 revealed that the frequency of the Hap1/2 genotype was significantly higher than that of the Hap2/2 genotype in the third litter (*p* < 0.05) and that the Hap1/1 genotype was not significantly correlated with litter size (*p* > 0.05).

### 3.7 CTSS bioinformatics analysis

The g.7413C → T mutation in the *CTSS* gene results in the substitution of serine by phenylalanine (p.S161F) and the g.8816A → T mutation results in the substitution of aspartic acid by tyrosine (p.N219Y). We analyzed the sequence conservation, function, stability, secondary structure and tertiary structure of CTSS proteins for mutant proteins.

#### 3.7.1 Sequence conservation analysis of CTSS proteins

**Figure 6:**
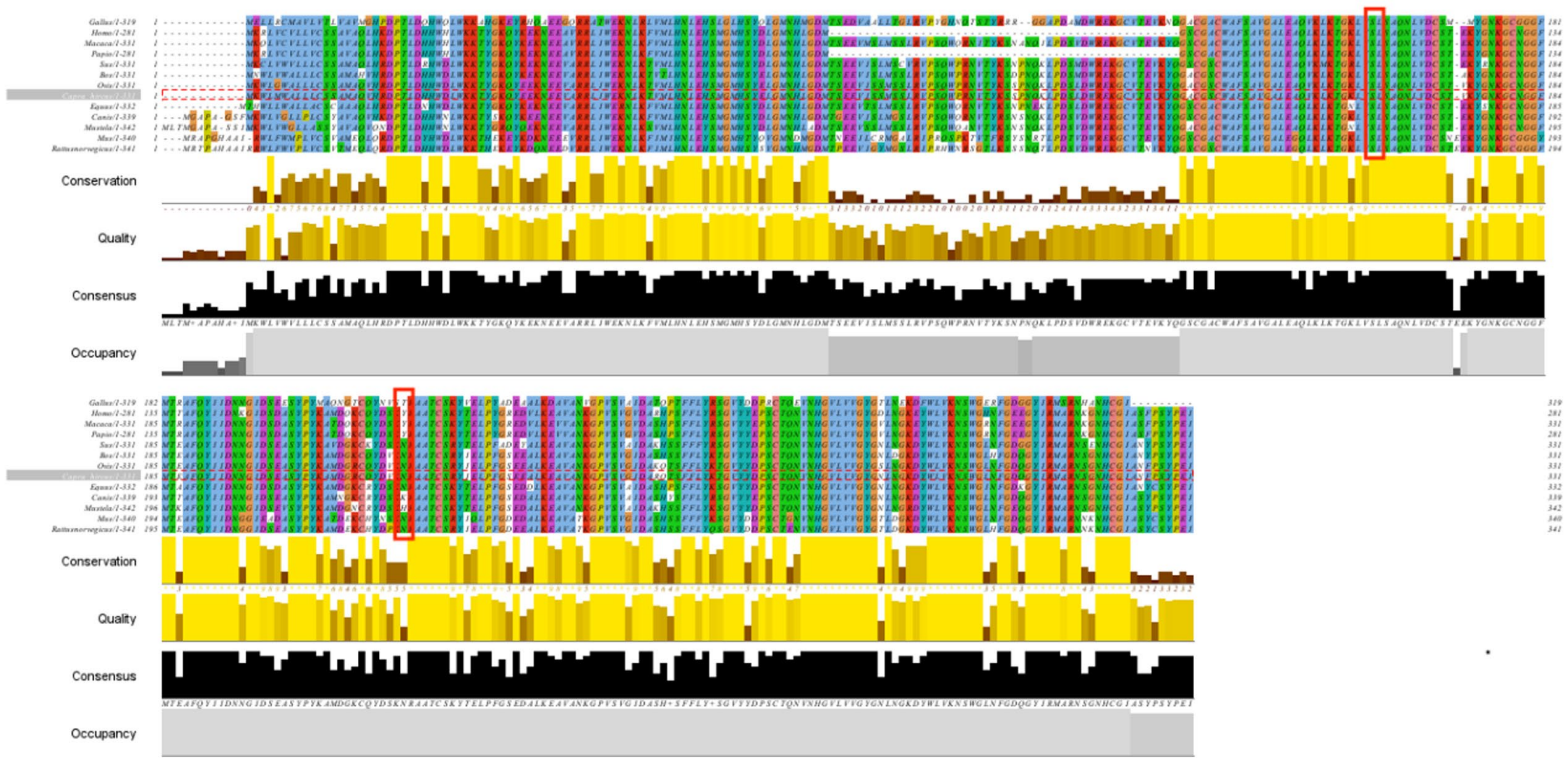
Protein sequence comparison of CTSS in 13 species.

The conserved p.S161F and p.N219Y sites in the CTSS amino acid sequence were analyzed using Cluster Omega online software, and the results are shown in Figure 6. p.S161F is highly conserved in 13 species, and p.N219Y is relatively conserved in even-toed ungulate species. The more highly conserved a site is, the more likely the mutation will have an effect on the structure and function of the protein.

#### 3.7.2 Effect of non-synonymous mutations on CTSS protein function

The effect of two nsSNPs on protein function was predicted to be neutral using PhD-SNP prediction software, with g.7413C > T scoring 3 on a scale of 0–9 and g.8816A > T scoring 2 on the same scale. According to SNAP2, is the SNPs are neutral, with scores of −38 and − 63, respectively, on a scale of −100 to 100.

#### 3.7.3 Effect of non-synonymous mutations on the stability of CTSS proteins

Analysis using I-Mutant 2.0 and MuPro showed that the p.S161F mutation increased the stability of the protein, the p.N219Y mutation decreased the stability of the protein, and the two mutations together reduced the stability of the CTSS protein (Tables 6–8).

**Table 6:** Association analysis between combined genotypes and the number of lambs produced in 1 ~ 3 litters of Qianbei sheep.

Combined genotype	First-born	Second-born	Third-born
Hap1/1	2.095 ± 0.501	2.257 ± 0.498	2.162 ± 0.683ab
Hap1/2	2.031 ± 0.740	2.125 ± 0.544	2.438 ± 0.669a
Hap2/2	1.714 ± 0.488	2.286 ± 0.488	2.000 ± 0.578b

Data in the table are compared in the same column within the same locus, different letters indicate significant differences between genotypes (*P* < 0.05) and the same letters indicate non-significant differences (*P* > 0.05).

**Table 7:** Prediction of the influence on CTSS protein function.

Prediction software	SNP locus	Amino acid mutation locus	Prediction results	Score
PhD-SNP	g.7413C>T	p. S161F	Neutral	3(0—9)
	g.8816 A>T	p. N219Y	Neutral	2(0—9)
SNAP2	g.7413C>T	p. S161F	Neutral	−38(−100 to 100)
	g.8816 A>T	p. N219Y	Neutral	−63(− 100 to 100)

A higher score indicates a greater impact on protein function.

**Table 8:** Protein stability prediction.

SNP locus	Amino acid mutation locus	I-Mutant 2.0	MuPro
		Free energy change (DDG)/(kJ▪mol^−1^)	Free energy change (DDG)/(kJ▪mol^−1^)
g.7413C>T	p. S161F	0.21	0.30
g.8816 A>T	p. N219Y	−0.43	−0.56

Delta delta G (DDG) < 0 means NSSNPS reduce protein stability, and delta delta G (DDG) > 0 indicates that NSSNPS protein stability.

#### 3.7.4 Effect of non-synonymous mutations on the secondary structure of CTSS proteins

The effects of p.S161F and p.N219Y mutations on the secondary structure of CTSS in goats were analyzed using Sopma, and the results are shown in Table 9. The secondary structures of the wild-type and mutant CTSS proteins contained four structures, namely, theα-helix, extended chain, β-turn and irregular curl, which accounted for 33.23, 17.52, 6.95, and 42.30% of the structures, respectively. The p.S161F and p.N219Y mutations resulted in a decreased proportion of extended chains and β-turns and an increased proportion of irregular curls.

**Table 9:** Prediction of the secondary structure of the CTSS mutant.

Type	Alpha helix	Extended strand	Beta turn	Random coil
CTSS-wild	33.23%	17.52%	6.95%	42.30%
CTSS-mutant	33.23%	16.92%	6.34%	43.50%

#### 3.7.5 Effect of non-synonymous mutations on the tertiary structure of CTSS proteins

**Figure 7:**
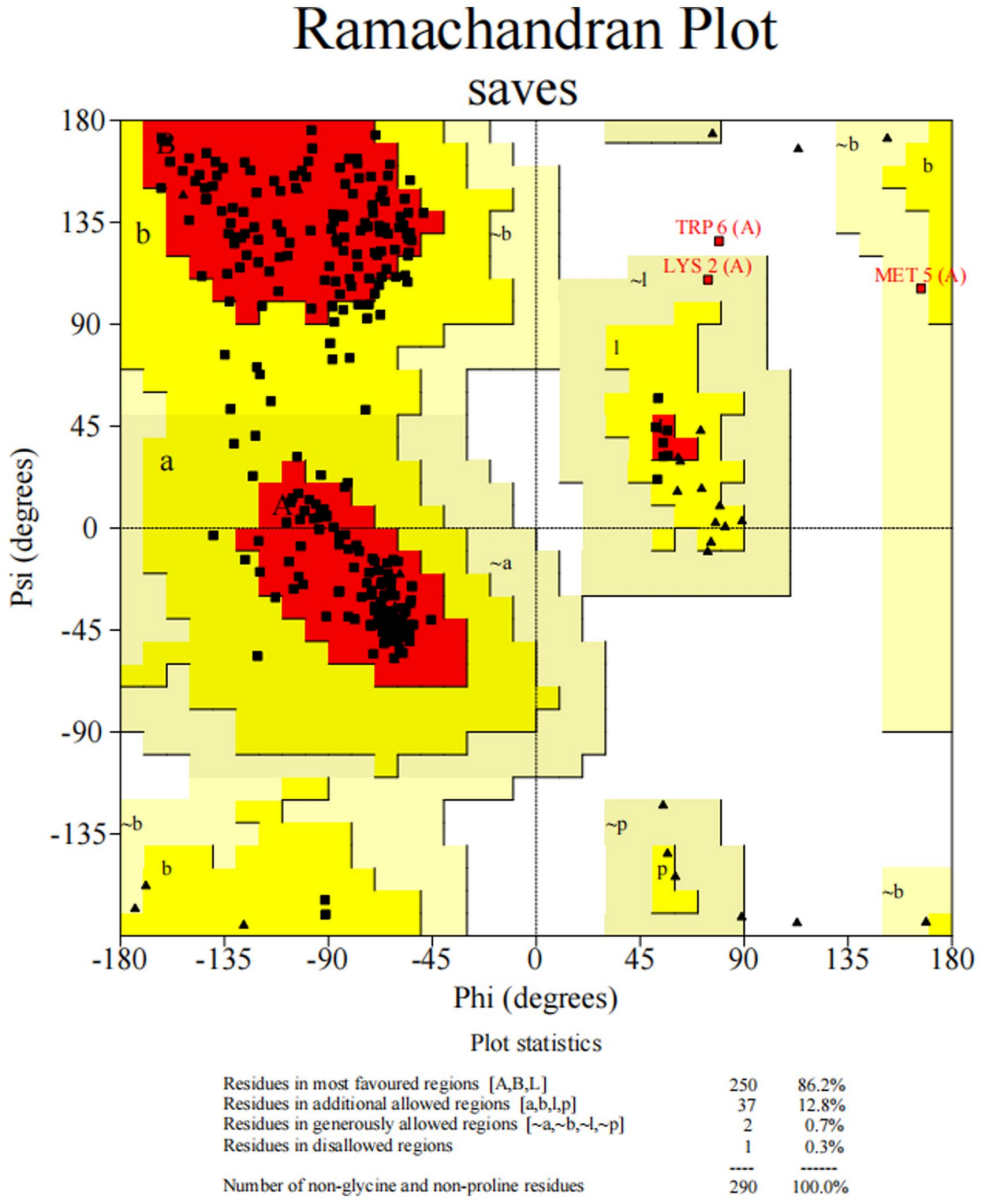
Predicted Raman profile of protein tertiary structure model reliability.

**Figure 8:**
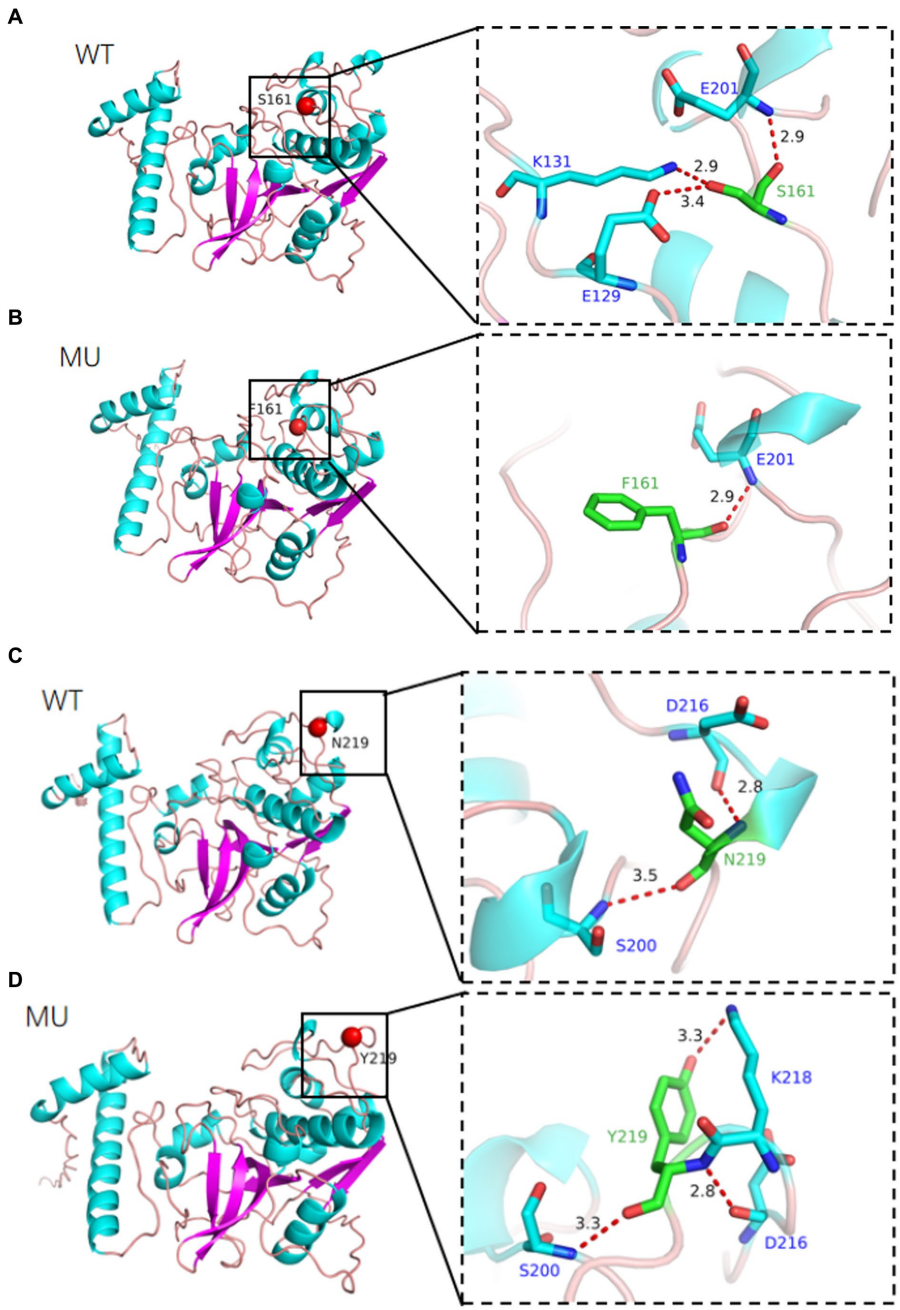
Simulation of the tertiary structure of CTSS protein, different colors represent different secondary structures, **(A)** indicates p.S161F site, **(B)** indicates p.N219Y site, (WT) wild-type, (MU) mutant type.

The tertiary structure of a protein is closely related to its function. We used AlphaFold2 to compare the 3D models of wild-type and mutant CTSS proteins at the p.S161F and p.N219Y loci, The model has 100% of amino acid residues in the reasonable region, and the protein structure obtained by the construction has high reliability and can be used as a template for subsequent studies (Figure 7). The constructed model is shown in Figure 8, the p.S161F site contains a non-polar positively charged serine substituted with a non-polar positively charged phenylalanine, and the p.N219Y site contains a polar uncharged aspartic acid substituted with a polar uncharged tyrosine. These two mutations resulted in altered amino acid interactions near the corresponding sites, leading to changes in the structure and function of the mutated protein but little effect on the 3D structure of CTSS.

## 4 Discussion

The lambing trait is one of important reproductive traits, and litter size is low heritability that is influenced by many factors, such as genetics, environment, management, and nutrition (27–29). Although there is a large body of research on the molecular basis of litter size in goats, the practical application of these findings is limited by the complexity of this quantitative trait (30, 31). *CTSS* polymorphisms are associated with acute atherosclerotic cerebral infarction (32). Mutations in the 5′-untranslated region of the *CTSS* gene were found to be strongly associated with feed conversion and average daily weight gain in Italian Large White pigs (33). In addition, *CTSS* is involved in the immune function pathway of high- and low-lambing rate populations in lake sheep, with critical effects on reproduction (34). Therefore, studying the *CTSS* gene will be beneficial to understand the variation in litter size in Qianbei Ma goats.

Qianbei Ma goats are resistant to adversity and disease, and retain the desirable traits of meekness, low odor, tender meat, and early sexual maturity (35). It is a valuable local breed in Guizhou Province, and the average litter size of it is lower than other domestic goat breeds (36, 37). To determine whether *CTSS* is a candidate gene for molecular breeding analysis in the high-reproductive performance Qianbei Ma goat population, we examined the expression of *CTSS* mRNA in the gonadal axis of single and multilamb ewes. In the single-lamb ewe population, *CTSS* expression was highest in the oviduct, whereas in the multilamb population, *CTSS* expression was highest in the uterus. *CTSS* is highly expressed in the sheep uterus; presumably, *CTSS* may be involved in endometrial remodeling and placenta formation in sheep (38, 39). *CTSS* induces increases in progesterone and estrogen levels in female rabbits to promote ovarian granulosa cell proliferation; estrogen and ovarian granulosa cells subsequently promote follicle development and ovulation (12, 39). The ovulation rate is an important determinant of litter size, while the uterus is critical for embryo implantation (15, 40, 41). Furthermore, *CTSS* expression underlies hormonal regulation in maternal tissues, is supportive of embryo implantation and is highly expressed in embryonic trophectoderm apposition sites and non-apposition sites (42, 43). Therefore, the upregulation of *CTSS* expression in the uterus of multilamb ewes improves lambing numbers in goats by affecting late embryo attachment. Therefore, *CTSS* may play an important role in reproduction in single and multilamb goats.

To investigate the regulatory mechanism of the *CTSS* gene in goat reproduction, we evaluated whether *CTSS* polymorphisms affect lambing traits in Qianbei Ma goats. After extraction of goat DNA, direct sequencing revealed that the genotype distribution of the SNP loci g.7413C → T, g.8816A → T and g.10193G → A did not deviate from Hardy–Weinberg equilibrium (HWE), while that of g.9191 T → G deviated from HWE. Further analysis revealed that all loci were moderately polymorphic (0.295 < PIC <0.310), which may be due to long-term artificial and natural selection (44). In addition, we analyzed the LD of the four mutant loci, and the analysis revealed that all of them were in strong LD (*r*^2^ > 0.33, *D*′ > 0.70). Correlation analysis showed that g.7413C → T, g.8816A → T, g.9191 T → G and g.10193G → A were all associated with litter size. In addition, the third litter number of lambs with Hap1/2 diploid (CTATTGGA) was significantly higher than that with Hap2/2 diploid (CCAATTGG). Therefore, *CTSS* expression may be closely related to the number of litters produced by goats. In the SNP analysis, we identified the g.7413C → T mutation in the *CTSS* gene as leading to the substitution of serine by phenylalanine at site 161 (p.S161F) and the g.8816A → T mutation as leading to the substitution of aspartic acid by tyrosine at site 219 (p.N219Y) of the CTSS protein. To elucidate the effect of this non-synonymous mutation on CTSS protein function, bioinformatics analysis revealed that compared with homologs in 12 other species, the p.S161F mutation was highly conserved in all 13 species, and p.N219Y was relatively conserved in even-toed ungulates and less conserved in other species (e.g., *Homo sapiens*, *Canis lupus familiaris*, *Gallus gallus*, and *Mustela putorius furo*). The mutation had a neutral effect on protein function; but, interestingly, the p.S161F mutation increased the stability of the protein, and the p.N219Y mutation decreased the stability of the protein. It has been shown that missense mutations that increase protein stability may also alter their function, that more stable proteins are more evolved and that mutations at the p.S161F and p.N219Y loci are consistent with speciation (45–47). Studies of the secondary and tertiary structures of the protein showed that mutations resulted in a decreased proportion of extended chains and β-turns and an increased proportion of irregular coiling. It has been found that a reduced β-turn angle and increased irregular coiling can improve a protein’s functional properties (48). Furthermore, our statistical analysis showed that the litter size was significantly greater in the g.7413C → T TT genotype group than in the corresponding CC and CT genotype group; At the g.8816 A → T mutation locus, the TT genotype gave birth to significantly more litter size than the AA genotype. Whether non-synonymous mutations in exons of this gene affect protein function by altering protein stability, thereby further affecting reproductive traits, needs to be determined by more in-depth studies.

## 5 Conclusion

In conclusion, our study showed that uterine *CTSS* mRNA expression levels in the multilambing ewe population were significantly higher than those in the single-lambing ewe population. Four SNPs loci in the *CTSS* gene of Qianbei ma goat were significantly associated with litter size, and the g.7413C → T and g.8816A → T mutations were non-synonymous mutations resulting in the substitution of serine 161 with phenylalanine and aspartate 219 with tyrosine in the CTSS protein. Bioinformatic predictions indicated that the p.S161F mutation in CTSS is highly conserved across 13 species, and p.N219Y mutation is relatively conserved in even-toed species; these mutations may significantly reduce the stability of the CTSS protein. These results suggest that the *CTSS* gene may be closely related to litter size in Qianbei Ma goats. These findings may provide new approaches for the breeding of high-fertility populations of Qianbei Ma goats.

## Data availability statement

The original contributions presented in the study are included in the article/supplementary material, further inquiries can be directed to the corresponding author.

## Ethics statement

The animal studies were approved by Guizhou University Subcommittee of Experimental Animal Ethics. The animal handling procedures were in line with the Chinese Animal Welfare Guidelines and were approved by the Animal Protection and Use Committee of Guizhou University, Guiyang, China (Approval number: EAE-GZU-2022-E030). The studies were conducted in accordance with the local legislation and institutional requirements. Written informed consent was obtained from the owners for the participation of their animals in this study.

## Author contributions

YZ: Conceptualization, Data curation, Methodology, Software, Validation, Writing – original draft, Writing – review & editing. XC: Conceptualization, Funding acquisition, Methodology, Project administration, Writing – review & editing. YR: Methodology, Software, Writing – review & editing. JC: Investigation, Writing – review & editing. WT: Investigation, Validation, Writing – review & editing. QJ: Validation, Writing – review & editing. KF: Writing – review & editing. WG: Review, Editing & supervision.
